# Role of Diabetes Mellitus in Heart Failure With Preserved Ejection Fraction: A Review Article

**DOI:** 10.7759/cureus.19398

**Published:** 2021-11-09

**Authors:** Okechukwu Mgbemena, Yixin Zhang, Gladys Velarde

**Affiliations:** 1 Cardiology, University of Florida College of Medicine, Jacksonville, USA; 2 Internal Medicine, University of Florida College of Medicine, Jacksonville, USA

**Keywords:** diastolic heart failure, diastolic dysfunction, metabolic changes and diabetes, diabetes type 2, diabetes treatment, heart failure with preserved ejection fraction

## Abstract

The pathophysiology of heart failure with preserved ejection fraction (HFpEF) is complex and poorly understood. There is a high prevalence of Diabetes Mellitus (DM) in patients with HFpEF, and the presence of DM has been shown to increase mortality of patients with HFpEF by 30%-50% even after adjustment for age, gender, hospital factors, and other patient characteristics. Since the prevalence of both entities is increasing worldwide, there is a need to explore their intricate relationship in order to elucidate potential management strategies to reduce the morbidity and mortality associated with this duo. In this review article, we explore the role of DM in the pathophysiology of HFpEF, ethnic and gender differences, and some therapeutic strategies in the management of patients with HFpEF and DM.

## Introduction and background

The incidence of type 2 DM and HFpEF has been on the rise over the last several decades. According to the World Health Organization (WHO), there were 422 million diabetic patients worldwide in 2014, which is a rise from 108 million in 1980. In 2014, more than 8.5% of adults aged 18 years and older had DM, and DM is the direct cause of death in 1.5 million patients [1]. The increasing incidence and complications of diabetes have led to significant morbidity and mortality, including blindness, kidney failure, myocardial infarctions (MI), and death. The pathophysiology of DM involves changes that worsen cardiac function at many levels. DM causes impaired cellular glucose uptake, increased serum glucose concentration, and uncoupling of mitochondrial oxidative phosphorylation mechanisms [2]. These subcellular and cellular changes lead to a toxic milieu with subsequent myocardial cell damage and abnormal cardiac relaxation patterns, which is the pathophysiologic hallmark of HFpEF [3].

HFpEF is a subtype of heart failure in which there is insufficient cardiac output for the metabolic needs of the body. The typical clinical features of DM associated with cardiac dysfunction include reduced ventricular compliance with increased systemic and pulmonary venous pressures and congestion despite preserved systolic function. This manifests as a clinical syndrome of volume overload associated with shortness of breath, orthopnea, paroxysmal nocturnal dyspnea (PND), rales/crackles, and at times with peripheral edema [4]. The clinical diagnosis of heart failure is made by two major or one major and two minor FRAMINGHAM criteria (Table 1) [4].

**Table 1:** FRAMINGHAM Criteria for diagnosis of heart failure

Major Criteria (Heart Failure diagnosis requires 1 or more major criteria)
Acute pulmonary edema
Cardiomegaly
Hepatojugular reflex
Neck vein distention
Paroxysmal nocturnal dyspnea or orthopnea
Pulmonary rales
Third heart sound (S3 gallup rhythm)
Minor criteria (heart failure diagnosis requires two or more minor criteria)
Ankle edema
Dyspnea on exertion
Hepatomegaly
Nocturnal cough
Pleural effusion
Tachycardia (heart rate >120 beats per minute)

Because all heart failure syndromes are inherently associated with diastolic dysfunction, the old term “diastolic heart failure” or “systolic heart failure” are being phased out in preference for categorization based on ejection fraction. There is heart failure with reduced ejection fraction (HFrEF, LVEF < 40%), heart failure with mid-range ejection fraction (HFmidRangeEF, LVEF 40%-50%), and heart failure with preserved ejection fraction (HFpEF, LVEF > 50%). The hallmark of HFpEF is diastolic dysfunction which entails impaired left ventricular (LV) relaxation and compliance. This leads to increased LV end-diastolic pressure and pulmonary capillary wedge pressure by invasive measurements. Non-invasively, diastolic dysfunction in HFpEF is diagnosed with three out of the four echocardiographic criteria: 1) decreased septal and lateral tissue doppler velocity (<7 and <10cm/s respectively); 2) elevated left atrial volume index > 34mL/m2; 3) average mitral inflow/tissue doppler velocity > 14; and 4) tricuspid regurgitation velocity > 2.8m/s [5].

**Figure 1:**
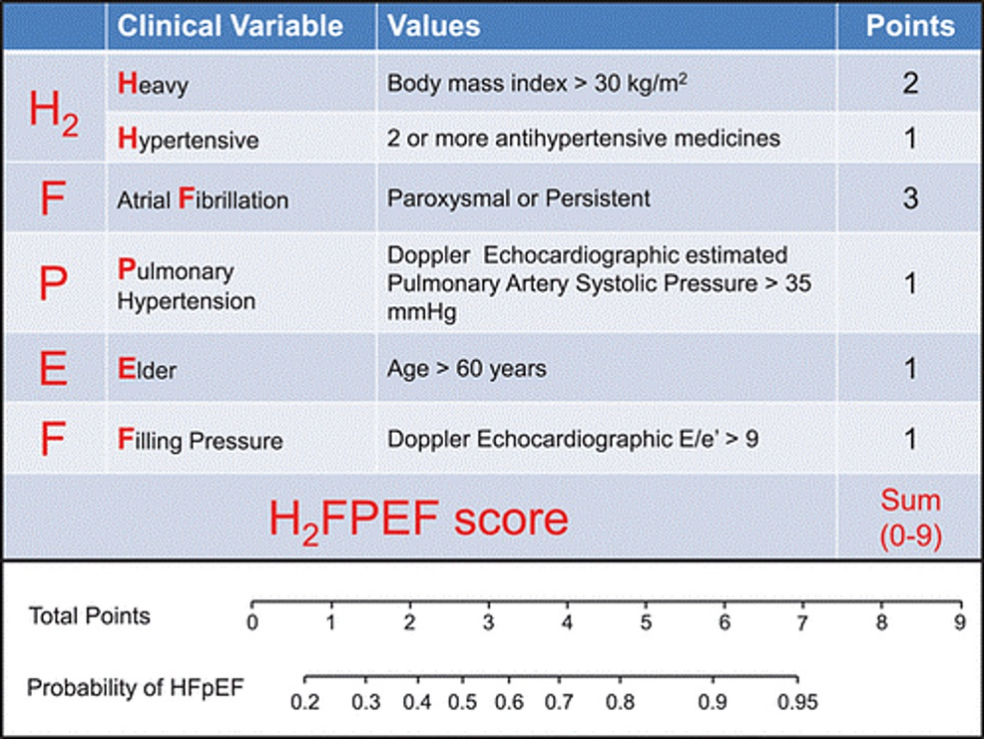
The H2FPEF score (Source: "A simple, evidence-based approach to help guide diagnosis of heart failure with preserved ejection fraction", Circulation

The diagnosis of HFpEF can be challenging in symptomatic but euvolemic patients, who present with non-specific symptoms such as dyspnea on exertion [6]. Because of this diagnostic challenge, the H2FPEF score (Figure 1) is becoming widely accepted as a tool to predict the diagnosis of HFpEF based on patient characteristics and echocardiographic findings. A low composite score (0-1) corresponds to a pretest probability of <20%, making the HFpEF diagnosis unlikely and suggesting non-cardiac causes for the symptoms. Conversely, a high score (6-9) is associated with a probability of HFpEF >90%, strongly suggesting HFpEF diagnosis [6]. 

The incidence of heart failure in the United States each year is about 650,000, and at least half of them have HFpEF [7]. Each year, the rate of emergency room visits due to HFpEF exacerbation is about 38.2% per 100 person-years. The cost of caring for patients with HFpEF is also on the rise and is estimated to be about $24,383 per patient per year. Despite advances in medicine, mortality for HFpEF patients has remained unacceptably high. According to Bhatia et al. [8], data pulled from 103 hospitals in Ontario, Canada, from 1999 to 2001 suggest a one year mortality rate of 22% due to HFpEF. Among patients with HFpEF, 25% have a coexisting diagnosis of DM, and its presence represents an independent risk factor for mortality, resulting in a 30%-50% increase in mortality when compared to patients with HFpEF without DM [9,10]. DM also confers a higher relative risk of cardiovascular death and heart failure hospitalizations in patients with HFpEF when compared to patients with HFrEF and DM [1]. Although DM is a well-known risk factor for atherosclerotic coronary artery disease, its role in the development of HFpEF is less established. While DM worsens diastolic dysfunction through microscopic changes associated with impaired myocardial relaxation, the mechanism of increased morbidity and mortality in HFpEF patients with DM also remains unclear [11,12].

Several hypotheses have been proposed, including macroscopic changes that result from altered glucose and free-fatty acid (FFA) metabolism in diabetic patients. The macroscopic remodeling that results from altered glucose and FFA metabolism include increased deposition and reduced degradation of extracellular collagen; interstitial fibrosis; myocyte hypertrophy; and intramyocardial microangiopathy. The absence of effective treatment for HFpEF represents an unmet medical need and has led to increased interest in this condition, its pathophysiology, and treatment strategies. In this literature review, we summarize the pathophysiologic mechanisms, the racial/ethnic and gender differences, and therapeutic implications.

## Review

**Figure 2:**
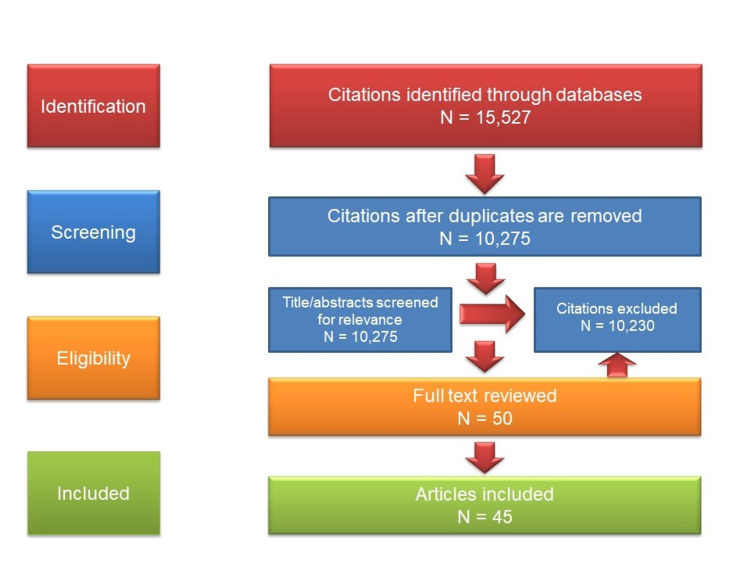
Literature review flow diagram for Role of Diabetes Mellitus in Heart Failure with preserved Ejection Fraction: A review article

A systematic review of the literature was performed using PubMed and Medline computerized databases. Published literature relating to DM and HFpEF was obtained using keywords “heart failure”, “heart failure with preserved ejection fraction”, “diastolic dysfunction”, “heart failure and diabetes”, heart failure with normal ejection fraction” in association with “Diabetes”, “Diabetes Mellitus” and “DM”. All literature was screened for appropriateness by title. There were 15,527 articles identified on the initial search. After duplicates were removed, the remaining 10,275 were screened by title for relevance to the subject matter. Out of the remaining publications, 10,225 were deemed irrelevant to the subject and were removed. Articles removed due to relevance addressed DM and other forms of heart failure other than HFpEF. Articles not written in the English language were also excluded. Abstracts without full articles were excluded. The remaining 50 articles were then reviewed in full and five were further excluded because they did not address the mechanistic relationship between DM and HFpEF or treatment options. Forty-five full articles were reviewed and included in this article. The article inclusion/exclusion process is shown in Figure 2. 

Mechanisms: genetic, transcriptional, and translational changes

At the transcription level, DM can lead to diastolic dysfunction by its action on cellular proliferation through interaction between insulin-like growth factors (IGFs) and growth hormones (GH) [13]. The RELAX trial studied the relationship between insulin-like growth factor-binding protein-7 (IGFBP7) and HFpEF, the result of the study concluded that higher baseline IGFBP7 was moderately correlated to worsening diastolic cardiac function with E velocity, higher left atrial volume index and elevated right ventricular systolic pressure [13]. IGFs and GH bind to the alpha subunit of IGF receptors on the cell surface of myocytes and subsequently causes a conformational change in the beta subunit of the receptor leading to activation of tyrosine kinase activity [14]. Tyrosine kinase then phosphorylates insulin receptor substrates (IRS), including 85 kDa regulatory subunit (p85) of phosphatidylinositol 3-kinase (PI 3-kinase), growth factor receptor-bound 2 (GRB2), and SH2-containing protein tyrosine phosphatase 2 (SHP2/Syp) [15]. These activated IRSs then activate genes, protein transcription, and translation, which are integral for cellular proliferation and hypertrophy. Abnormal cellular hypertrophy and cellular matrix deposition then lead to impaired relaxation of cardiac myocytes and LV diastolic dysfunction [16].

In addition to the above transcriptional and translational changes, there are other possible mechanisms, including upregulation of fetal gene programming involving an increase in beta-myosin heavy chain gene (MHG) and deregulation of fast-contracting isoforms, all of which lead to an impaired rate of myocardial relaxation and worse diastolic dysfunction in HFpEF [17].

Mechanisms: multi-protein interactions and cellular changes

Due to altered glucose and free fatty acids (FFA) metabolism, there is increased utilization of FFA in myocytes of patients with diabetes and HFpEF. Because the metabolism of FFA utilizes more oxygen compared to the metabolism of glucose, there is increased oxidative stress and the formation of oxidative free radicals within myocytes that lead to cell injury and diastolic dysfunction [12]. Over time, these oxidative free radicals can induce apoptotic signals within myocytes leading to cell death and worsening diastolic function.

In addition to the formation of cardiotoxic oxidative free radicals in patients with HFpEF, DM also promotes the formation of advanced glycosylation end-products (AGEs), activation of polyol, and NADP-pathways [18,19]. These changes further increase oxidative stress and free radical damage within myocytes causing, worsening myocardial injury.

Chronic oxidative stress leads to myocardial fibrosis and increased intracellular and extracellular matrix deposition. Several pathways involved in the downregulation of matrix degradation are also activated, further causing a worsened imbalance between matrix deposition and degradation. LV diastolic dysfunction and HFpEF are partly due to this imbalance [20].

DM is associated with diffuse microangiopathy that is not readily apparent on traditional coronary angiography, which detects macrovascular disease. Due to coronary microangiopathy and associated endothelial dysfunction, there is impaired oxygen delivery and utilization by myocytes. There is also a decrease in coronary flow reserve associated with blunted effects of adenosine and endogenous nitrates [21-22]. These cumulative changes lead to myocardial ischemia and diastolic dysfunction.

**Figure 3:**
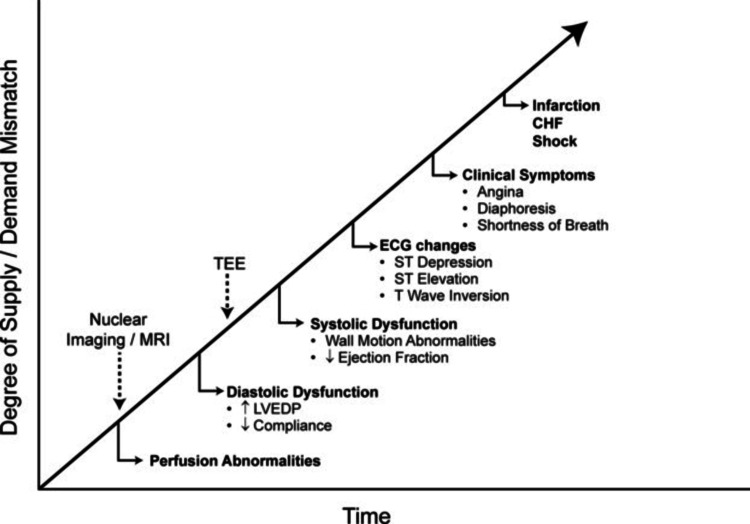
Ischemic cascade - progressive pathophysiologic changes as myocardial oxygen supply mismatch progresses (Sourced from Cleveland Clinic Center for Medical Art & Photography)

Macrovascular disease is a well-known contributor to diastolic dysfunction and HFpEF. Diabetes leads to accelerated macrovascular disease that worsens atherosclerotic build-up seen in obstructive coronary artery disease (CAD) [23]. With the progression of obstructive CAD, diastolic dysfunction is one of the earliest signs of demand-supply mismatch of oxygen in cardiac myocytes after intracellular perfusion abnormalities (Figure 3). If myocyte oxygen demand-supply continues to worsen beyond diastolic dysfunction, patients typically progress to develop systolic dysfunction marked by a decrease in LVEF before electrocardiographic changes and clinical symptoms become apparent [23]. Myocardial infarction and cardiogenic shock are end-stage manifestations of severe oxygen demand/supply mismatch. 

Mechanisms: neurohormonal dysregulation

Diabetes is related to a wide array of several neurohormonal dysregulations that contribute to adverse outcomes in patients with HFpEF. Through increased angiotensinogen released from the adrenal glands and upregulation of cardiac angiotensin II receptors, DM is associated with advanced hypertensive heart disease and diastolic dysfunction [2]. Hyperglycemia and hyperinsulinemia are also associated with central autonomic neuropathy and increased peripheral salt retention, leading to further impairment of diastolic function.

Mechanisms: heightened inflammatory milieu

Increasing evidence exists to support the role of the heightened inflammatory milieu present in DM with progression of diastolic dysfunction. DM is associated with increased concentration of serum inflammatory markers such as C-reactive protein (CRP), soluble suppression of tumorgenesis-2 (sST2), galectin-3, C-terminal propeptide of procollagen type I (PICP), N-terminal propeptide of procollagen type III (PIIINP), matrix metalloproteinase-9 (MMP-9) and tissue inhibitor of matrix proteinase-1 (TIMP-1) [24]. It has been shown that patients with HFpEF and DM have higher levels of (PIIINP), galectin-3 (Gal-3), and TIMP-124. Interestingly, circulating levels of Gal-3 are associated with the degree of myocardial fibrosis and can predict re-hospitalization and all-cause mortality in HF. In addition, Gal-3 is an excellent marker for the detection of earlier cardiac remodeling [25]. These increased inflammatory markers are postulated to lead to myocardial fibrosis and worse outcomes in patients with HFpEF and DM. It is worth noting that in 2013, the American College of Cardiology Foundation/American Heart Association's (ACCF/AHA) heart failure guidelines recommended the use of two myocardial fibrosis markers, galectin-3 and soluble ST2, for risk stratification with Class IIb recommendations [26].

Racial and ethnic factors

**Figure 4:**
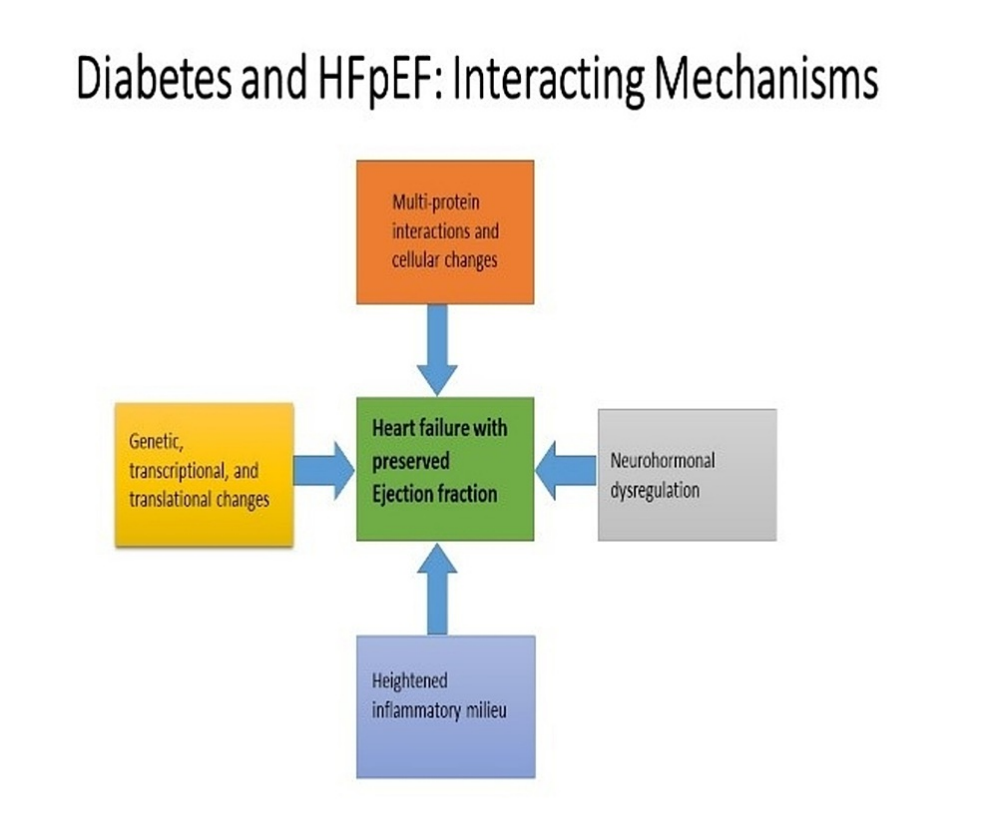
Interacting Mechanisms

The lifetime risk for new incidence of HFpEF is higher in white than in black patients, but black patients have worse heart failure readmissions compared to white patients [27]. A sub-analysis of the TOPCAT trial (treatment of preserved cardiac function heart failure with an aldosterone antagonist) in North and South America demonstrated a higher risk for the primary outcome (HR, 1.34; 95% confidence interval, 1.06-1.71; P=0.02) and first HF hospitalization (HR, 1.51; 95% confidence interval, 1.167-1.97; P=0.002) for black patients compared to white patients [27]. This higher rate of HFpEF readmission for black patients persists even after adjusting for socioeconomic status, hospital factors, and other patient characteristics [28,29]. Although there are increased heart failure hospitalizations in black patients, there is lower one-year mortality among Black, Hispanic, and Asian patients compared to white patients [29]. The explanation for this paradox remains elusive. The interacting mechanisms of diabetes and heart failure with preserved ejection fraction is depicted in Figure 4.

Gender differences

The lifetime incidence of HFpEF is similar in both men and women, but the female sex is associated with worse outcomes, including higher rates of HFpEF hospitalizations [30]. The rationale for worse outcomes in female patients may be partly due to hormonal factors [31]. Sex hormones, such as testosterone and estrogen, are key contributors to physiological differences between men and women in general.

**Figure 5:**
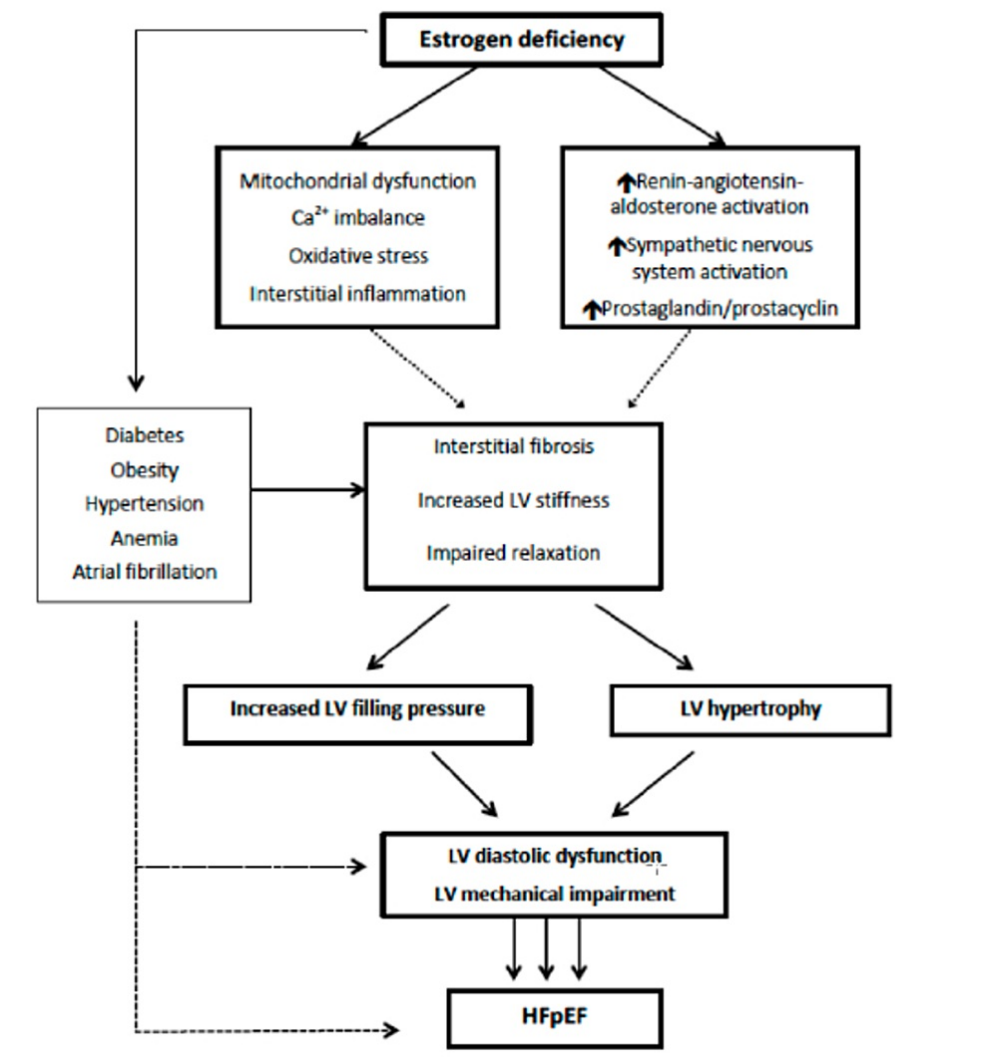
Estrogen deficiency on LV remodeling and dysfunction (Tadic, Marijana, et al.)

Several epidemiological and associative studies highlight the role of estrogens in diabetic heart disease as a sex- and age-dependent event [30,31]. However, research on estrogen-dependent intracellular signaling within the female myocardium is still ongoing. Interestingly, these hormones also play an indirect role in the pathophysiology of HFpEF, as noted by Tadic, Marijana, et al. (Figure 5). There is a vicious cycle between estrogen deficiency and insulin resistance [30]. Menopausal estrogen deficiency, through a variety of mechanisms like loss of protective miRNA and augmentation of X-linked miRNA expression, may lead to mitochondrial dysfunction, calcium imbalance, heightened oxidative and inflammatory milieu, consequently giving rise to a female-specific cardiovascular etiology of HFpEF [30]. Estrogen deficiency also triggers an increase in renin-angiotensin-aldosterone activity. These changes lead to interstitial fibrosis, increased LV stiffness, and impaired LV relaxation, all of which are the hallmark of HFpEF [30-32]. The relationship between female gender and worse clinical outcomes in HFpEF has not been well-elucidated and is an area of intense research [30,32].

Therapeutic implications

There is currently no consensus regarding the most effective preventive or therapeutic approaches to treat HFpEF, let alone those with concomitant DM. For that reason, guidelines recommend diuretics for fluid removal and symptom relief, and management of associated comorbidities (e.g., hypertension and obesity). Currently, the management of high blood glucose levels in patients with DM and CV risk and CVD is tailored to minimize the risk of CV complications, with metformin as first-line therapy [33]. Few oral hypoglycemic agents have been shown to improve heart failure outcomes, especially heart failure hospitalizations. Medications associated with improved heart failure outcomes include biguanides and SGLT2 inhibitors (Table 2) [34].

**Table 2:** Summary of oral hypoglycemic trials/studies in HFpEF

Trial/Study	Class (Drug)	Outcome
Facila et al.; Aguilar et al.; Masoudi et al.; Romero et al.	Biguinides (metformin)	Improved heart failure outcomes improved MACE
UKPDS; ABC; ACE	Alpha-glucosidase inhibitors	Neutral heart failure outcomes
EMPA-REG OUTCOME; EMPEROR-Preserved; CANVAS; DECLARE-TIMI 58	SGLT2-Inhibitors (empagliflozin and dapagliflozin)	Improved heart failure outcomes Improved MACE
SAVOR-TIMI 53; EXAMINE trial; TECOS trial	DPP-4 inhibitors (saxagliptin; alogliptin; sitagliptin)	Neutral heart failure outcomes (except saxagliptin which is associated with worse outcome for heart failure hospitalization)
PROactive; RECORD	Thiazolidinediones (pioglitazone and rosiglitazone)	Worse heart failure outcomes
NAVIGATOR trial	Glinides (nateglinide)	Neutral heart failure outcomes
UKPDS 33	Sulfonylureas	Neutral heart failure outcomes
Cycloset Safety trial	D2-dopamine agonist (bromocriptine)	Neutral heart failure outcomes
ORIGIN trial	Insulin (glargine)	Neutral heart failure outcomes
ELIXA trial; LEADER trial; SUSTAIN-6; EXSCEL trial	GLP-1 receptor agonist (lixisenatide; liraglutide; semaglutide; exenatide)	Neutral heart failure outcomes

Other oral hypoglycemic agents have neutral cardiovascular outcomes and does not show to improve or worsen ischemic and heart failure outcomes. These neutral classes of medications include DPP-4 inhibitors [43,44]; insulin (glargine) [45,46]; alpha-glucosidase inhibitors [47-49]; glinides [50]; D2-dopamine agonists [46]; GLP-1 agonist [51-53]; and sulfonylurea [54].

Several trials and meta-analyses have established that metformin, the only publicly available biguanide, is safe, efficacious, and is associated with improved cardiovascular outcomes in heart failure patients, making metformin first-line therapy for the treatment of DM in HFpEF patients [35-38]. A recent systematic review and meta-regression analysis suggest that the use of metformin in patients with HFpEF is associated with reduced mortality in HFpEF and HFrEF after adjustment for heart failure therapies such as angiotensin converting enzymes inhibitors (ACEI) and beta-blockers (BB). Significantly greater protective effects were seen in patients with HFpEF compared to HFrEF [35-37]. Concomitant use of metformin and insulin were also associated with a reduction in mortality of HFpEF. 

Recently, SGLT2 inhibitors, especially empagliflozin, have also been shown to improve diastolic dysfunction and heart failure outcomes in both rodent models and humans with HFpEF through a reduction in wall stress by decreasing preload and altered hemodynamics [34]. Trials demonstrating efficacy or non-inferiority of SGLT2 in heart failure include the empagliflozin, cardiovascular outcomes, and mortality in type 2 diabetes (EMPA-REG OUTCOME) trial [39]; canagliflozin cardiovascular assessment study (CANVAS) [34,40], and multicenter trial to evaluate the effect of dapagliflozin on incidence of cardiovascular events (DECLARE-TIMI 58) [40]. The recently published EMPEROR-Preserved is the first phase 3 clinical trial that exclusively enrolled patients with HF and ejection fraction of >40% to meet its primary outcome. About half of the patients in this trial had DM. In this study, empaglifozin led to a 21% relative reduction in the rate of CVD death or HF hospitalizations in these patients [39-41]. This represents the first agent to unequivocally meet its primary outcome and benefit patients with HFpEF with or without DM. 

It is important to note that the use of thiazolidinediones (TZDs) in heart failure patients has been shown to increase heart failure hospitalizations [41]. The prospective pioglitazone clinical trial in macrovascular events (PROACTIVE) and rosiglitazone evaluated for cardiac outcomes and regulation of glycemia in diabetes (RECORD) trials [42] demonstrated that increase in heart failure hospitalizations and worse outcomes in patients taking TZDs is due to fluid and sodium retention associated with TZDs so this group of oral hypoglycemics is generally avoided in heart failure patients regardless of LVEF.

## Conclusions

HFpEF and DM commonly co-exist, and the presence of DM independently and significantly increases mortality in patients with HFpEF. The presence of DM in HFpEF patients worsens diastolic dysfunction through several mechanisms involving altered glucose and FFA metabolism; transcriptional and translational changes; increased oxidative stress through actions of free radicals; neurohormonal dysregulation; heightened inflammatory milieu, and microvascular/macrovascular alterations and injury. There are racial differences in HFpEF patients with DM. The lifetime risk for incident HFpEF is higher in non-blacks than in black patients, but black patients have worse heart failure readmissions compared to non-blacks. Females are also associated with worse outcomes, including the rate of HFpEF hospitalizations, although the prevalence of HFpEF is comparable in men and women. Therapeutic strategies for the management of diabetes that have been shown to improve cardiovascular outcomes include biguanides and SGLT2 inhibitors, while TZDs are associated with adverse heart failure outcomes and should be avoided in heart failure patients. Other medication classes, including DPP-4 inhibitors; insulin (glargine); alpha-glucosidase inhibitors; glinides; D2-dopamine agonists; GLP-1 agonist; and sulfonylurea are neutral to heart failure outcomes.
